# QM/MM Energy Decomposition Using the Interacting Quantum Atoms Approach

**DOI:** 10.1021/acs.jcim.1c01372

**Published:** 2022-02-25

**Authors:** Roberto López, Natalia Díaz, Evelio Francisco, Angel Martín-Pendás, Dimas Suárez

**Affiliations:** † Departamento de Química y Física Aplicadas, Universidad de León, Facultad de Biología, Campus de Vegazana s/n, 24071 León (Castilla y León), Spain; ‡ Departamento de Química Física y Analítica, Universidad de Oviedo, Facultad de Química, Julián Clavería 8, 33006 Oviedo (Asturias), Spain

## Abstract

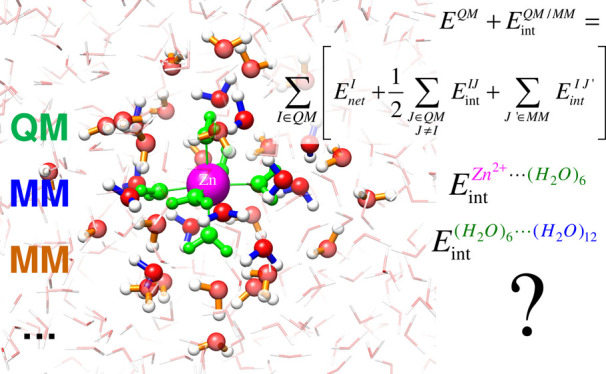

The interacting quantum atoms (IQA) method decomposes the quantum mechanical (QM) energy of a molecular system in terms of one- and two-center (atomic) contributions within the context of the quantum theory of atoms in molecules. Here, we demonstrate that IQA, enhanced with molecular mechanics (MM) and Poisson–Boltzmann surface-area (PBSA) solvation methods, is naturally extended to the realm of hybrid QM/MM methodologies, yielding intra- and inter-residue energy terms that characterize all kinds of covalent and noncovalent bonding interactions. To test the robustness of this approach, both metal–water interactions and QM/MM boundary artifacts are characterized in terms of the IQA descriptors derived from QM regions of varying size in Zn(II)– and Mg(II)–water clusters. In addition, we analyze a homologous series of inhibitors in complex with a matrix metalloproteinase (MMP-12) by carrying out QM/MM–PBSA calculations on their crystallographic structures followed by IQA energy decomposition. Overall, these applications not only show the advantages of the IQA QM/MM approach but also address some of the challenges lying ahead for expanding the QM/MM methodology.

## Introduction

Certainly, pure quantum mechanical (QM) or hybrid quantum mechanical/molecular mechanical (QM/MM) methods are indispensable tools in biomolecular modeling given that, in principle, they are systematically improvable, provide a high degree of transferability, and, in most cases, they include all the effects required for a proper description of chemical reactions, noncovalent interactions, ligand chelation to metals, etc. In this way, QM methods are capable of overcoming some of the limitations of the physically based MM methods that generally do not incorporate an explicit representation of electronic effects like polarization and charge transfer. Since high-level QM calculations are only affordable for systems of up to a few hundred atoms, hybrid QM/MM methods are usually employed in order to treat at the QM level the region comprising the active site residues and substrate molecules involved in enzymatic mechanisms or the ligand and nearby receptor residues that are critical for binding affinity.^1,2^ Additionally, both QM and QM/MM calculations can be useful for MM parameterization tasks^3,4^ as well as in the development of more accurate MM potentials^5^ for the fast simulation of biomolecules.

The usefulness of QM or QM/MM calculations in biomolecular modeling may be augmented by carrying out an energy decomposition analysis (EDA), which aims to ascertain the nature and type of interactions among the molecular components as well as to rationalize their stabilizing or destabilizing roles.^6^ There is no unique method to decompose QM energies and, consequently, many EDAs have been proposed to provide alternative decompositions of relative energies into physically meaningful additive terms. In fact, EDA methodologies are routinely applied to decompose all kind of energies including those obtained using highly correlated QM methods.^7^ For example, a recent study using the local energy decomposition (LED) technique on large cluster models has demonstrated that the protein–ligand QM binding energies can be split into fragment-pairwise contributions characterizing in detail the binding hot spots.^8^ However, the partitioning of QM/MM energies including environmental effects are still relatively scarce and, therefore, it would be interesting to expand the applicability of the various EDA techniques in order to treat large systems described by QM/MM Hamiltonians.

The decomposition of QM/MM energies would be also useful to assess both the truncation and overpolarization effects that limit their accuracy. In general, the reliability of the QM/MM calculations improves with increasing the size of the QM region and decreasing the polarity of the groups located at the QM–MM boundary.^9^ However, there are still important issues in the QM/MM methodology for which a QM/MM EDA could yield atomic and/or fragment-based energy descriptors useful to characterize, among others, the optimal choice of the QM region,^10,11^ the electrostatic interaction between QM and MM atoms,^12^ and the handling of the QM–MM covalent bonds. Therefore, the implementation of the QM/MM EDA protocols would satisfy a twofold goal by contributing to measure the energetic impact of specific groups/interactions as well as to provide relevant information about specific QM/MM methodological problems.

As mentioned above, multiple energetic partitions have been developed that, in most cases, give energy contributions whose physical meaning is framed within the reference adopted to define the interacting components. Thus, the symmetry-adapted perturbation theory (SAPT)^13^ and its many variants make use of a perturbative approach to differentiate the distinct nature of the weak noncovalent interactions among molecular species. On the other hand, the family of orbital-based EDAs^14^ exploits a stepped scheme to calculate various energy terms (e.g., electrostatic, Pauli repulsion, orbital, and dispersion) with respect to reference electronic state(s) that, in turn, may correspond to radical species representing covalently bound fragments, a reference molecular geometry, or isolated molecules. Similarly, the LED method employs local representations of the occupied and virtual orbital spaces, as built by the linear-scaling local correlation methods, in order to divide the correlation energy into intra- and interfragment contributions and classify the double excitation contributions into different physical components.^7^ As a consequence, the range of applicability of the orbital-based EDA and LED methods is much less restricted than that of SAPT, thus allowing the analysis of strong interactions and intramolecular effects.

Considering the decomposition of the QM/MM energies of large systems, the definition of the orbital EDA/LED methods within the Hilbert space expanded by MOs/LMOs is in contrast with the real-space character of the MM force fields that typically collect different atomic contributions that, in turn, would hamper the partitioning of the hybrid QM···MM interactions. As an alternative, the interacting quantum atoms (IQA) method,^15,16^ which relies on real-space partitioning into the attraction (atomic) basins (Ω_*I*_) of the gradient field of the QM electron density and thereby provides self-atomic energies *E*(Ω_*I*_) and diatomic energies *E*(Ω_*I*_,Ω_*J*_), seems a priori a suitable EDA technique to incorporate QM/MM effects. This expectation is supported by the IQA capability to dissect the classical and exchange-correlation energies either in chemical bonds or in noncovalent interactions.^17,18^ In addition, using DFT (and HF) densities, IQA is applicable to medium-sized systems in combination with the Grimme’s D3 potential,^19,20^ which yields pairwise dispersion energies that complement the diatomic *E*(Ω_*I*_,Ω_*J*_) terms.^21^ Furthermore, it has been shown that the IQA net atomic energies can easily absorb the electrostatic continuum-solvent effects, allowing thus the partition of solvation energies into effective atomic and group contributions.^22^

Taking into account the potential interest of the QM/MM EDAs and the favorable features of the IQA method, the goal of this work is to demonstrate the viability and usefulness of the IQA-based decomposition of QM/MM energies. To this end, we briefly describe the theoretical details of IQA and its extension to accomplish the decomposition of QM/MM energies, including also solvent effects as described by the Poisson–Boltzmann method. Then, the adequacy of this approach is shown by carrying out a series of test calculations in two different cases of study. First, we will examine metal/solvent contacts considering the biologically relevant metal ions Zn(II) and Mg(II)^23^ as well as the water molecules located in their closest hydration shells. In these metal···water QM/MM calculations, the sequential increase in the number of solvent molecules included in the QM region will permit us to assess the usefulness of the IQA terms in tracking the energy impact of QM/MM boundary artifacts. The second application analyzes a metalloenzyme/ligand complex featuring strong metal–ligand contacts. In particular, we selected the matrix metalloproteinase MMP-12 enzyme, which is a well-characterized zinc-peptidase enzyme involved in a number of physiological and pathological conditions,^24^ to analyze the strength of enzyme/inhibitors contacts at the catalytic site. Finally, we will comment on the potential advantages and drawbacks of the IQA QM/MM calculations.

## Theory

### IQA Decomposition of QM (DFT-D3) Energies

According to the original formulation of the IQA approach,^15,16^ the ab initio QM energy (*E^QM^*) of a molecular system is decomposed by integrating the first-order reduced density matrix (RDM), ρ_1_(**r_1_**,**r_1_′**), and the second-order RDM, ρ_2_(**r_1_**,**r_2_**), within the topological atomic basins (Ω_*Ι*_) derived from the charge distribution ρ(**r**). The energy decomposition comprises both atomic and diatomic terms
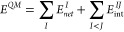
1where *E*_*net*_^*I*^ is the atomic net energy that includes the electronic kinetic energy and the potential energy due to nucleus–electron (*ne*) attractions and electron–electron repulsions (*ee*) within Ω_I_. The *E*_int_^*IJ*^ terms collect various potential energies (*nn*, *en*, *ne*, and *ee*) involved in the interaction between atoms *I* and *J*. The ρ_2_(**r_1_**,**r_2_**) density can be split according to ρ_2_(**r_1_**,**r_2_**) = ρ_1_(**r_1_**) ρ_2_(**r_2_**) + ρ_*xc*_(**r_1_**,**r_2_**), where ρ_1_(**r_1_**) ρ_2_(**r_2_**) represents a non-correlated product of densities while ρ_*xc*_(**r_1_**,**r_2_**) stands for the exchange-correlation (*xc*) density. Accordingly, it is feasible to compute an electrostatic component of the interaction energy along with an exchange-correlation contribution such as *E*_int_^*IJ*^ = *E*_*ele*_^*IJ*^+ *E*_*xc*_^*IJ*^.

By grouping half the interaction energy of atom *I* with the remaining net energy, we define its additive energy
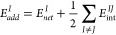
2so that the sum of all the *E*_*add*_^*I*^ terms reproduces the total energy *E^QM^*. The individuality of each pair is maintained in its definition so that the particular properties of atoms and pairs are inherited by *E*_*add*_^*I*^. This procedure yields also a thermodynamic limit-compatible partition of the energy into additive components similar to that provided by the energy of a unit cell in a crystal, which can be rigorously found as the limit of the total energy of the system per cell as the size of the system grows.^25^ From another point of view, additive energies allow to decrease the complexity of the IQA rationale. Instead of examining an *O*(*N*^2^) number of interactions to rationalize a given behavior, we focus on an *O*(*N*) set of quantities that condense, or trace out, the quadratic number of interactions in large systems while remaining exactly rigorous as we approach the thermodynamic limit.

The IQA approach can also decompose the QM energies calculated with DFT methods. The lack of a DFT second-order reduced density ρ_2_(**r_1_,r_2_**) is circumvented by computing effective atomic *xc* energies, *E*_*xc*_^*I*, *DFT*^, following similar prescriptions as those of the HF method. Then, scaled intra- and interatomic *xc* energies are derived so that the total DFT energy is exactly recovered by summing the scaled *xc* energies. In this work, we employ the scaling technique developed by Martín-Pendás et al.,^26^ which has been shown to give satisfactory results.

The IQA partitioning of HF or DFT energies can be readily combined with pairwise dispersion corrections such as the third-generation (D3) correction using the Becke–Johnson rational damping function.^20^ The dispersion interaction energies *E*_*disp*_^*IJ*^ are merely added to the rest of the IQA diatomic terms leading to a D3-corrected IQA decomposition^21^
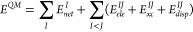
3

The IQA decomposition admits the grouping of atomic terms into fragment contributions (e.g., functional groups and molecules). Thus, a fragment decomposition of a molecular aggregate constituted by two moieties A and B involves
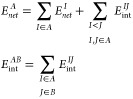
4where *E*_*net*_^*B*^ can be calculated analogously to *E*_*net*_^*A*^. For practical purposes, we use the IQA acronym to refer to the atomic analysis, whereas for its fragment version, the term interacting quantum fragments (IQF) is preferred. Using D3-IQF, the formation (or binding) energy of a molecular aggregate constituted by two fragments A and B (A + B → AB) is divided into fragment deformations and interfragment interactions as
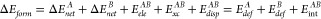
5where the deformation term *E_def_* stands for the variation of the net energy of the fragment, which collects both the intra- and interatomic IQA energies belonging to the corresponding fragment. The interfragment interaction energy collects the electrostatic (*E*_*elec*_^*AB*^), exchange-correlation (*E*_*xc*_^*AB*^), and empirical dispersion (*E*_*disp*_^*AB*^). At this point, it may be worthy to remark that IQA interaction energies should not be confused with relative energies, such as Δ*E_form_*, that measure the stability of a given complex with respect to the separate fragments.

### Amber Force Field

Although different MM methods could be coupled with IQA, here, we focus on the Amber force field,^27,28^ which in its simplest form uses the following expression to calculate the MM energy
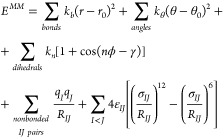
6

In this equation, we distinguish between the bonded and nonbonded terms. The former include the harmonic terms for bond stretching and angle bending, which account for the fluctuations of the bond-length *r* and bond-angle θ, respectively, with respect to their reference values (*r*_0_ and θ_0_) under the influence of the force constants (*k_b_* and *k*_θ_). The usual Fourier-series expansion modulated by the parameters *k_n_* and γ plays a critical role in describing the rotational barriers around the dihedral angles ϕ. Note that formally, the stretching, bending, and torsional terms correspond to 2-, 3-, and 4-body effects. Of more particular interest for the QM/MM methodologies are the nonbonding interactions between atoms located at different molecules or separated by at least three consecutive bonds. These are described by pairwise (2-body) potentials varying with the interatomic distance *R_IJ_*. Thus, electrostatic contributions are merely computed with the Coulomb law involving partial atomic charges *q_I_* and *q_J_*, typically obtained as fitting parameters to the QM electrostatic potential. The van der Waals interactions (including short-range repulsions as well as dispersion and polarization attractions) are described by the empirical Lennard–Jones potential, which includes two parameters corresponding to the depth of the potential (ε_*IJ*_) and the distance where the potential is null (σ_*IJ*_).

### IQA Partitioning of the QM/MM Interaction Energy

Assuming that there is no covalent linkage between the QM and MM regions, the corresponding QM–MM Hamiltonian includes the nonbonded van der Waals (*vdW*) interactions among QM and MM atoms and the electrostatic interaction between the QM charge density and the partial charges of the MM atoms. To perform a consistent treatment of electrostatic interactions within the IQA framework, the electronic embedding of the QM region is required, thus allowing the explicit polarization of the QM charge density due to the presence of the point charges on the MM atoms. The nonbonding QM–MM interaction energy *E*_int, *nb*_^*QM*/*MM*^ is then computed using the Coulomb law and the Lennard–Jones potential as
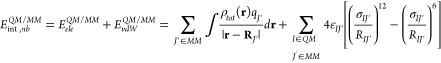
7where ρ_*tot*_(**r**) is the total charge density of the QM region, including both the QM electronic density ρ_*e*_(**r**) and the nuclear charges *Z_I_* at positions *R_I_* (i.e., ). Of course, *E*_*vdW*_^*QM*/*MM*^ is readily decomposable as a sum of diatomic contributions (i.e., , the MM atom in *E*_*vdW*_^*IJ*′^ being denoted by the primed index). Similarly, within the IQA approach, the equivalent decomposition of *E*_*ele*_^*QM*/*MM*^ into atomic contributions is straightforward
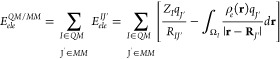
8where each *E*_*ele*_^*IJ*′^ is obtained by the monoelectronic integration of the QM density within the Ω_*I*_ basin of atom *I* and the double sum runs over the QM and MM atoms.

The presence of covalent linkages between QM and MM atoms does not pose special problems in order to partition the bond–angle–torsion (BAT) terms connecting QM and MM atoms given that these terms are readily split and assigned to atomic additive energies (see below). In addition, we assume that H-link atoms are used to treat the QM–MM boundary as implemented in the Amber package^29^ so that electrostatic interactions between all MM atoms (excluding MM atoms directly bonded to a QM atom) are calculated for all QM atoms, including the link atom. In this manner, the electrostatic interactions of the MM link atoms are replaced by those of the QM H-link atoms.

### QM/MM Energy Decomposition

The total energy of a QM/MM system can be written as
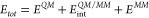
9where *E^QM^* and *E^MM^* collect all the energy contributions arising from the separate QM and MM regions, respectively, while *E*_int_^*QM*/*MM*^ would include both the nonbonded interactions and the BAT terms between QM and MM atoms. *E_tot_* can be expressed as a sum of additive atomic energies, , as long as *E*_int_^*QM*/*MM*^ is evenly split between the QM and MM atoms. Using IQA, the additive energy of a given QM atom contains its net energy (*E*_*net*_^*I*^), half the diatomic interaction energies with other QM atoms (*E*_int_^*IJ*^), and half the corresponding QM–MM pairwise energy (*E*_int_^*IJ*′^)
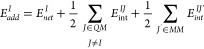
10

Analogously, the additive energy of a MM atom (*E*_*add*_^*I*′^) brings together the bonded and nonbonded terms in which the atom *I′* is involved
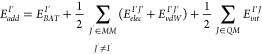
11where *E*_*BAT*_^*I*′^ accounts for one-half of the bond energies, one-third of the angle energies, and one-fourth of the torsion energies (the BAT contributions to the QM–MM *E*_*int*_^*I* ′ *J*^ terms are distributed in the same fashion).

In many practical applications of nonpolarizable QM/MM methods, one is interested in the calculation of relative energies (e.g., for the formation of a host–ligand complex) involving fixed geometries. In this case, the IQA-based decomposition of the relative energies implies the cancellation of the MM (and QM–MM) BAT terms and of the nonbonded terms between MM atoms. For the sake of simplicity, it is then convenient to omit the purely MM terms (*E*_*BAT*_^*I*′^, *E*_*elec*_^*I* ′ *J*′^, and *E*_*vdW*_^*I* ′ *J*′^) and restrict our analysis to the partitioning of the QM energy plus the QM–MM interaction (i.e., *E^QM^* + *E*_int_^*QM*/*MM*^). This energy, which may be termed simply as the QM/MM energy *E*^*QM*/*MM*^, can be also expressed as a sum of additive energies over the QM atoms
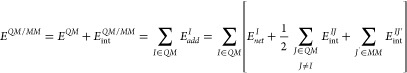
12

Note, however, that each QM–MM pairwise energy (*E*_int_^*IJ*′^) in this equation is entirely ascribed to the QM atom *I*, whereas the QM additive energy defined in eq 10 absorbs only one-half of *E*_int_^*IJ*′^. The applications reported in this work consider only the decomposition of *E*^*QM*/*MM*^ energies, and the corresponding additive energies are given in accordance with eq 12.

### Inclusion of Solvent Effects

In general, the estimation of binding affinities or other energies using QM/MM calculations largely benefits from the inclusion of solvent effects as described by solvent continuum models.^30^ Although some proposals for coupling self-consistent reaction field (SCRF) methods with QM/MM Hamiltonians have been reported,^31^ the implementation of QM/MM SCRF methods is still scarce. Alternatively, it is feasible to combine single-point QM/MM energies with the electrostatic solvation energy estimated by means of implicit solvent methods like Poisson–Boltzmann (PB),^32^ which represents the solute molecule in terms of a set of atomic partial charges and parameterized radii. Thus, PB determines the electrostatic reaction field potential Φ_*RF*_ exerted by the solute through the numerical solution of the Poisson equation^33^ and expresses the electrostatic contribution to the solvation free energy as a sum of atomic contributions involving the product of partial charges and the Φ_*RF*_ values at the atomic positions
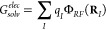
13so that the IQA additive energies *E*_*add*_^*I*^ can be directly combined with the atomic *q_I_*Φ_*RF*_(**R**_*I*_)contributions.

The electrostatic PB solvation energy is usually complemented with the nonpolar parts due to cavity formation and van der Waals interactions between the solute and the solvent molecules. Following the proposal of Tan et al.,^34^ the cavity (repulsive) *G*_*solv*_^*cav*^ and the dispersion (attractive) *G*_*solv*_^*disp*^ energies can be estimated separately using the following empirical expressions:
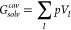
14where *p* is a solvent pressure parameter and *V_I_* is the volume enclosed by the solvent-accessible surface (SAS) of atom *I*, and
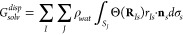
15where the attractive dispersion energy is derived from the solvent density and the surface integrals over the SAS of atom *J* (*S_J_*) of the empirical Θ(**R**_*Is*_) function defined on the SAS of atom *I*, ***n***_*s*_ being the outward normal vector associated to the SAS element σ_***s***_. Hence, *G*_*solv*_^*cav*^ is constructed as a sum of atomic contributions, while *G*_*solv*_^*disp*^ is obtained by a double sum of nonsymmetrical diatomic contributions (*G*_*solv*_^*disp*, *IJ*^), which, for the sake of energy decomposition, are conveniently symmetrized as *G′*_*solv*_^*disp*, *IJ*^ = (*G*_*solv*_^*disp*, *IJ*^ + *G*_*solv*_^*disp*, *JI*^)/2. As a result, an effective atomic contribution to the solvation free energy can be defined as
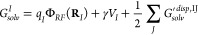
16

In this way, the gas-phase QM/MM atomic additive energies in eq 12 can be complemented with the *G*_*solv*_^*I*^ terms in order to fully decompose at the atomic level the QM/MM energy in aqueous solution, *E^QM^* + *E*_int_^*QM*/*MM*^ + *G_solv_*.

## Computational Section

### Mg(II)/Zn(II)–Water Clusters

The molecular clusters of the hydrated Mg(II)/Zn(II) ions were built from structures generated by molecular dynamics (MD) simulations of the metal cations in explicit solvent. Thus, the Mg(II)/Zn(II) ions were solvated by a 25 Å spherical cap of ∼5400 TIP3P waters.^35^ MM parameters for the cations solute species were taken from the IOD-TIP3P set.^36^ Energy minimization and MD calculations of the hydrated systems were carried out using the sander program included in the AMBER18 package.^37^ The water molecules were initially relaxed by means of 1000 conjugate-gradient steps. Subsequently, a 200 ps MD trajectory was computed in which only the cap water molecules were allowed to move. The solvent cap was restrained at the 25 Å boundary by a harmonic potential with a force constant of 0.125 kcal/(mol Å^2^). The time step of the MD simulations was 1.0 fs, and the SHAKE algorithm constrained all the bond lengths at their equilibrium values. A nonbond pairlist cutoff of 15.0 Å was used, and the temperature was maintained at 300 K using the Berendesen’s algorithm.

The last MD snapshot was selected to carry out a series of single-point QM/MM calculations in which the metal ion and an increasing number *n* of the closest water molecules were included in the QM region (*n* = 6, 18, 42, 90, and 186 waters). The QM subsystem was described with the hybrid B3LYP^38,39^ method in combination with the triple-ζ cc-pVTZ basis set^40−42^ in which the set of *g* functions for Zn were not included in order to diminish the computational cost of the IQA calculations (test calculations were performed with and without the *g* functions, showing that their impact on the IQA descriptors is minimal; see Table S2). The MM water molecules were described with the TIP3P potential. The single-point QM/MM calculations were performed with the sander program coupled with the ORCA 4.0.1 program^43^ and using no cutoff.

### Selection of the MMP-12 Complexes

**Scheme 1:**
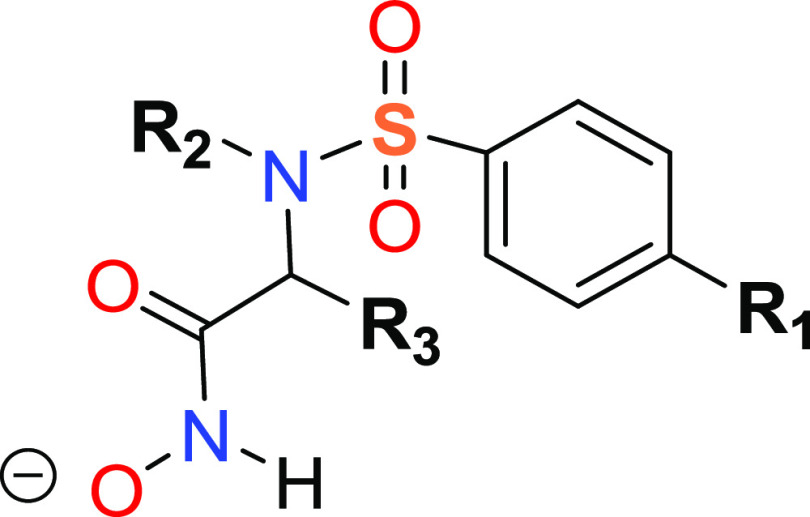
Scaffold for the Hydroxamate-Based MMP-12 Inhibitors

The MMP-12 complexes studied in this work were selected on the basis of the availability of experimental binding data (*K*_I_) and high-resolution crystallographic structures (Table 1). We focused on inhibitors bearing hydroxamic groups because these zinc-binding groups (ZBGs) give close contacts with the catalytic Zn ion. Thus, we considered the homologous series of MMP-12 inhibitors developed by Bertini et al.^24^ that are characterized by a sulfonamide scaffold (structures hs1, hs3, hs4, hs5, hs6, hs7, z79, and nhk; see Scheme 1 and Table 1). The variations in the binding affinities of these ligands, which range in the nM−μM interval at pH 7.2, have been rationalized in terms of small structural changes in specific enzyme–ligand contacts.^24^

**Table 1:** Inhibitor IDs, PDB IDs, Resolutions (Å), and Experimental Binding Data of the MMP-12 Complexes Used in this Study

ID	R_1_	R_2_	R_3_	PDB	*K*_I_ (nM)
hs1	-OCH_3_	-CH_2_CHOHCH_2_OH	H	3F15 (1.70 Å)	7.88
hs3	-OCH_3_	-H	-(D)CH_2_OH	3F16 (1.16 Å)	5.91
hs4	-H	-Ph	-H	3F17 (1.10 Å)	2.36
hs5	-F	-CH_2_CH_2_OH	-H	3F18 (1.13 Å)	39.5
hs6	-F	-H	-H	3F19 (1.13 Å)	65.1
hs7	-H	-H	-H	3F1A (1.25 Å)	61.1
z79	-OCH_3_	-H	-H	3LK8 (1.80 Å)	19.7
nhk	-OCH_3_	-CH_2_CH_2_OH	-H	3NX7 (1.80 Å)	7.88

To perform single-point QM/MM calculations on the X-ray structures of the complexes formed between the catalytic domain of MMP-12 (158 residues from *N*-Gly_106_ to *C*-Gly_263_) and the selected inhibitors, only the coordinates of the protein atoms, the Zn(II)/Ca(II) ions and the ligand atoms were taken from the corresponding PDB files (see Table 1). The subsequent edition of the systems was done with the tools included in the AMBER18 suite of programs (tleap, antechamber, sqm, sander, etc.).^37^ The ff14SB version of the all-atom Amber force field was used to represent the protein residues.^44^ Hydrogen atoms were added by tleap considering the standard protonation state of the acid/basic residues, except that of Glu_219_, which was modeled in its neutral form (see below). The two Zn(II) ions and the three Ca(II) ions were described by nonbonding parameters that reproduce experimental ion–oxygen distance values and coordination numbers of the first solvation shell.^36^ Amber GAFF parameters^45^ were assigned to the ligand molecules by means of the antechamber program. The same program also assigned QM charges to the ligand atoms, which were derived at the B3LYP/6-31G(d) level of theory^46,47^ with the RESP methodology,^48^ the QM calculations being carried out with Gaussian09 (revision B.01).^49^

The hydroxamic groups of the MMP-12 inhibitors shown in Scheme 1 are weak acids with predicted p*K_a_* values of 8–9. However, previous QM/MM calculations on MMP/inhibitor complexes have shown that the mode of binding of ligands bearing zinc-binding groups, such as hydroxamic, is consistent with a negatively charged ZBG and a neutral carboxylic group for the conserved Glu side chain,^50,51^ which is largely favored by the solvent and enzyme environment. Thus, the Glu_219_ residue was modeled in its neutral protonation state, while the hydroxamic group of the ligands was negatively charged.

To better describe the structure of the Glu_219_-COOH···hydroxamate contacts, the X-ray structures were partially relaxed by means of QM/MM geometry optimizations. The QM/MM geometry optimizations were carried with the sander program available in the AMBER18 package, which provides a QM/MM interface with the Terachem program.^52,53^ The QM region included the side chains of the Zn_1_-bound histidine residues (His_218_, His_222_, and His_228_) and that of Glu_219_, the catalytic Zn_1_ ion, and the ligand atoms. H-link atoms were inserted by sander at the corresponding Cα–Cβ bonds. The QM region was described at the B3LYP/6-31+G* level of theory (including D3 dispersion corrections), while the rest of the protein atoms were treated with the ff14SB force field with no cutoff. During the QM/MM geometry optimization, only the protein H atoms and the QM atoms were allowed to move until the root mean square of the Cartesian elements of the gradient was less than 0.02 kcal/(mol Å) (2 × 10^–5^ in au). These QM/MM calculations further confirmed the stability of the Glu_219_-COOH···hydroxamate contacts.

### QM/MM–PBSA Energy Scorings

Single-point QM/MM–PBSA energies (*G*) were evaluated on the partially relaxed X-ray structures. For each MMP-12/inhibitor complex, we estimated the binding energy between the enzyme and the inhibitor by taking the corresponding difference of the *G* values: Δ*G* = *G*(*cmplx*) – *G*(enz*) – *G*(inh*). In this expression, the asterisk superscript means that the *G* energies of the enzyme and inhibitor molecules are evaluated using their geometries in the complex. The resulting Δ*G* values should be considered as physically based scoring functions as they ignore both distortion effects and proton rearrangement upon inhibitor binding, as well as configurational entropy changes. The QM/MM–PBSA energies are computed according to the following equation
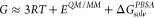
where *E^QM/MM^* is the gas-phase QM/MM energy, the 3*RT* contribution is due to six translational and rotational degrees of freedom, and Δ*G*_*solv*_^*PBSA*^ is the solvation energy, which consists of both polar and nonpolar contributions.

The QM/MM calculations were carried with the sander program adopting the MM potential that was previously built by tleap from the ff14SB/GAFF parameters. The QM region included again the side chains of the Zn_1_-bound histidine residues (His_218_, His_222_, and His_228_) and that of Glu_219_, the catalytic Zn_1_ ion, and the ligand atoms. The B3LYP/cc-pVTZ(*−g*)^38,39^ level was used in combination with the D3 dispersion corrections choosing the Becke–Johnson damping function.^19,54^ The QM/MM calculations were driven by the sander program selecting the QM/MM interface for the ORCA 4.0.1 package.^43^

The gas-phase QM/MM energy *E* was combined with a solvation energy term (Δ*_solv_G*) estimated by means of the PB method.^55^ For the QM atoms, we derived the atomic charges from the B3LYP/cc-pVTZ(−*g*) electrostatic potentials using a grid-based method (CHELPG charges)^56^ by means of the orca_chelpg utility program. Since the QM electrostatic potential is obtained by means of QM/MM calculations, the resulting CHELPG charges include in an effective way some polarization effects induced by the surrounding MM atoms. For the MM atoms, the ff14SB charges were used. We employed the pbsa program to solve the nonlinear Poisson–Boltzmann equation^33^ on a cubic lattice by using an iterative finite-difference method. We selected a grid spacing of 0.33 Å, null ionic strength, and the solute and solvent dielectric constant values ε_*sol*_ = 1 and ε_*solv*_ = 80, respectively. The dielectric boundary was built as the contact surface between the modified Bondi atomic radii of the solute (as assigned by the tleap program) and the radius (1.4 Å) of a water probe molecule. The total PBSA energy also included the implicit nonpolar terms (dispersion and cavity) according to the model of Tan et al.^34^

### IQA Calculations

The IQA decomposition of the QM energies was performed with a modular version of the PROMOLDEN program^57^ that is being developed in our laboratory. In this version, the program reads the point charges representing the MM region in order to compute the QM–MM electrostatic interaction term (*E*_int, *elec*_^*QM*/*MM*^) using the same integration algorithm that is employed for computing the electron–nucleus interaction terms *V_en_* of the QM region.^15^

The IQA quantities are numerically integrated by PROMOLDEN over finite and irregular integration domains using ultrafine angular and radial grids in atomic spherical quadratures.^15,58^ We employed integration settings that represent a compromise choice between computational cost and accuracy. Thus, a β-sphere around each atom was considered (i.e., a sphere completely contained inside the atomic basin), with a radius equal to 60% the distance of its nucleus to the closest bond critical point in the electron density. High-quality Lebedev angular grids were used with 5810 and 974 points outside and within the β-spheres of heavy atoms, respectively (3890 and 590 points for hydrogen atoms). Euler–McLaurin radial quadratures were employed with 512 and 384 radial points outside and inside the β-spheres of heavy atoms, respectively (384 and 256 points for H atoms). The largest value of the radial coordinate in the integrations was 15.0 au for heavy atoms (10.0 au for H atoms). Maximum angular moments, λ_max_, of 10 and 6 were assigned to the Laplace and bipolar expansions of 1/*r*_12_ outside and within the β-spheres.

To speed up the computation of the IQA terms, the modular PROMOLDEN version uses localized MOs and employs the multipolar approach for computing selected interatomic exchange-correlation (*xc*) energies. The LMOs were computed with the Pipek–Mezey algorithm^59^ as implemented in the ORCA 4.0.1 package. For each atomic basin, Ω_*I*_, a subset of LMOs {ϕ_*i*_^*LMO*^}_*I*_ is built by requiring that their diagonal contribution to the atomic overlap matrix (∫_Ω_*I*__|ϕ_*i*_^*LMO*^|^2^*d*τ) is greater than 10^–6^ au. The calculations of the IQA *E*_*net*_^*I*^ terms are done using the subset {ϕ_*i*_^*LMO*^}_*I*_ for each basin. For the calculation of the diatomic electrostatic *E*_*ele*_^*IJ*^ terms, the two LMO sets of the pair of basins (i.e., {ϕ_*i*_^*LMO*^}_*I*_ ∪ {ϕ_*j*_^*LMO*^}_*J*_) are required in order to describe the charge density in each basin. However, a smaller set of LMOs is needed for the calculation of the nonclassical *E*_*xc*_^*IJ*^ energy given that only those LMOs that appear in both sets (i.e., {ϕ_*i*_^*LMO*^}_*I*_ ∩ {ϕ_*j*_^*LMO*^}_*J*_) contribute to the integration of the *xc* interactions. The multipolar *xc* approximation^60^ at high-order (*L* = 10) is activated for 1 – *k* (*k* > 4) intramolecular interactions provided that the interatomic *R_IJ_* distance is greater than 5.0 au. For *R_IJ_* > 17 au, the *E*_*xc*_^*IJ*^ values are neglected. In previous work,^17^ it has been found that these approximations do not compromise the conclusions of the IQA calculations because their impact on the total numerical error is quite small.

## Results and Discussion

### QM/MM Calculations on the Hydrated Zn(II)/Mg(II) Systems

**Figure 1:**
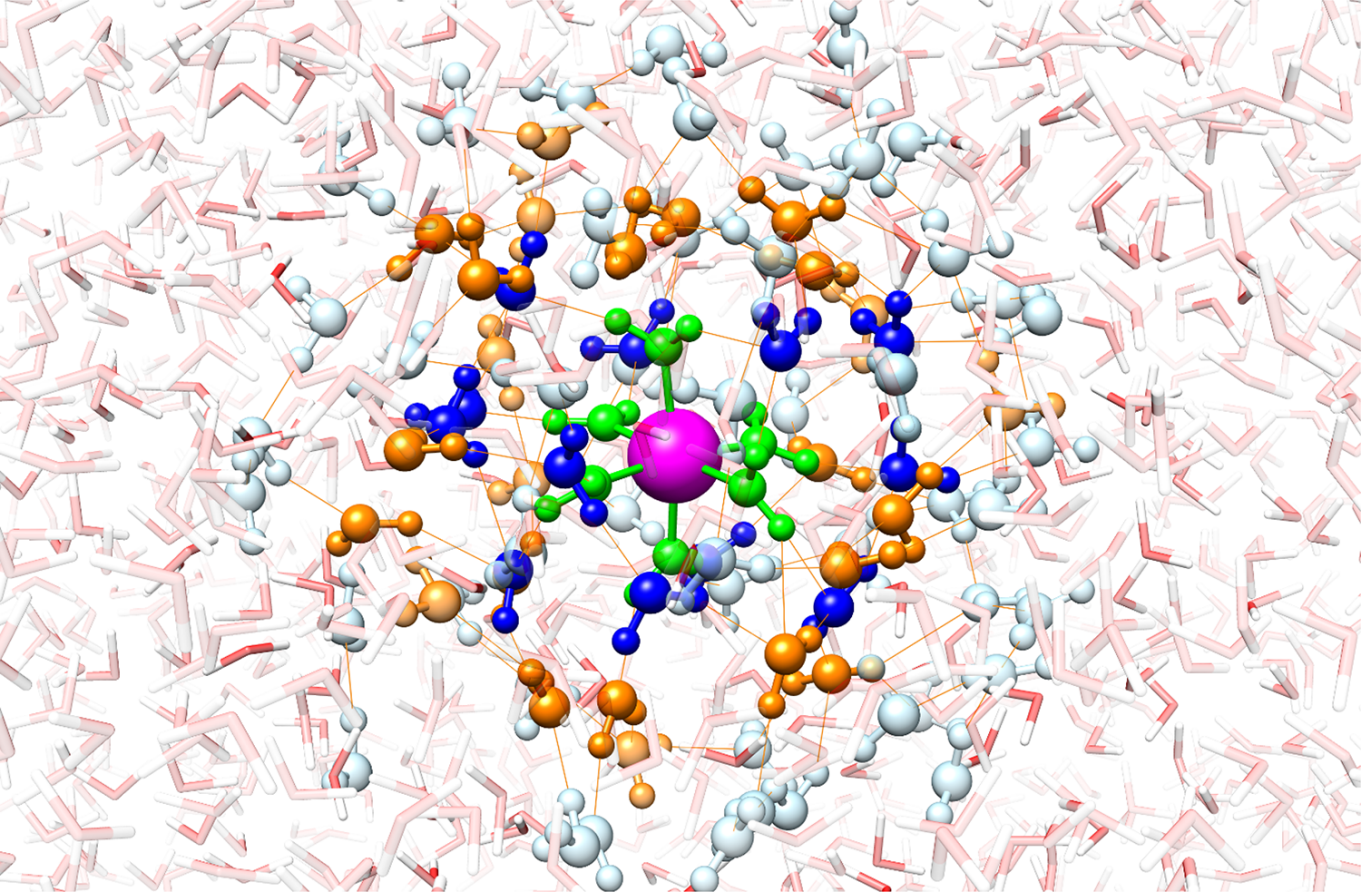
Model of the hydrated Zn(II) ion examined in this work. The metal cation (in magenta) is shown as a vdW sphere, while the four closest shells of water molecules are shown in ball-and-stick representations of various colors (green–blue–orange–light blue), denoting shell membership.

Single-point QM/MM calculations at the B3LYP/cc-pVTZ//TIP3P level were performed on the Zn(II)/Mg(II) ions surrounded by a 25 Å spherical cap of water molecules (see Figure 1). In these models, the first hydration shell of the Zn(II) and Mg(II) ions, which have very similar ionic radii (0.88 and 0.86 Å), corresponds to an octahedral coordination environment. Only the closest waters to the metal ions, which were grouped into five shells containing 6, 12, 24, 48, and 96 molecules, were included in the QM region.

The absolute QM/MM energies of the hydrated metals were obtained for various choices of the QM region (*M* – Wat_*n*_^*QM*^ with *M* = Zn(II)/Mg(II) and *n* = 0, 6, 18, 42, 90, and 186 being the total number of QM waters), followed by their decomposition using the IQA protocol without the dispersion D3 terms. The main results are summarized in Table 2, which collects the water → metal charge transfer (Δ*q*) and selected IQA/IQF energy components (Δ*E*_*add*_^*M*^, Δ*E*_*net*_^*M*^, *E*_int_^*QM*^ = *E*_*ele*_^*QM*^+ *E*_*xc*_^*QM*^, *E*_*ele*_^*QM*/*MM*^ ) that refer to the metal atom and its interaction with the surrounding waters. Thus, Δ*E*_*add*_^*M*^ and Δ*E*_*net*_^*M*^ stand for the additive and net energy of the metal cation in the cluster, which are given as relative quantities with respect to its isolated gas-phase counterparts. The *E*_int_^*QM*^, *E*_*ele*_^*QM*^, and *E*_*xc*_^*QM*^ terms correspond to the IQF energies that comprise the interaction of the metal with the QM waters, while *E*_*ele*_^*QM*/*MM*^ accounts for the electrostatic interaction of the central ion with the MM waters. Given that the short-range *E*_*xc*_^*QM*^ converges rapidly with the size of the QM region (see below) and its high computational cost, the IQA calculations for the largest cluster models (*n* = 90 and 186) evaluated only the net energies of the metal and its electrostatic pairwise energies.

**Table 2:** Water → Metal Charge Transfer (Δ*q* in *e*^–^), Change of the IQF Additive Atomic Energies (Δ*E*_*add*_^*M*^ in au), and Energy Components (Δ*E*_*net*_^*M*^, *E*_*ele*_^*QM*/*MM*^, *E*_*int*_^*QM*^, *E*_*ele*_^*QM*^, and *E*_*xc*_^*QM*^ in au) for the Metal Ion and its Interaction with the Surrounding Waters in Each System Studied^b^

QM subsystem	Δ*q*	Δ*E*_*add*_^*M*^	Δ*E*_*net*_^*M*^	*E*_*ele*_^*QM*/*MM*^	*E*_*int*_^*QM*^	*E*_*ele*_^*QM*^	*E*_*xc*_^*QM*^	*E*_*int*_^*QM*^*_+_E*_*ele*_^*QM*/*MM*^	*E*_*ele*_^*QM*^*_+_E*_*ele*_^*QM*/*MM*^
Mg(II)	0.000	–1.2126	0.0010	–1.2136				–1.2136	–1.2136
Mg – Wat_6_^*QM*^	0.209	–0.9754	–0.0884	–0.5497	–0.6746	–0.5352	–0.1394	–1.2243	–1.0849
Mg – Wat_18_^*QM*^	0.212	–0.8953	–0.0892	–0.3393	–0.9337	–0.7931	–0.1406	–1.2730	–1.1324
Mg – Wat_42_^*QM*^	0.212	–0.8646	–0.0893	–0.2673	–1.0160	–0.8751	–0.1408	–1.2833	–1.1425
Mg – Wat_90_^*QM*^	0.212	–0.8344	–0.0849	–0.2105	–1.0780	–0.9372	–0.1408^a^	–1.2975	–1.1477
Mg – Wat_186_^*QM*^	0.212	–0.7246	–0.0849	–0.0702	–1.1384	–0.9973	–0.1408^a^	–1.2086	–1.0677
Zn(II)	0.000	–1.2426	0.0058	–1.2485				–1.2485	–1.2485
Zn – Wat_6_^*QM*^	0.493	–1.1083	–0.3025	–0.4700	–0.6716	–0.2704	–0.4012	–1.1416	–0.7404
Zn – Wat_18_^*QM*^	0.497	–1.0670	–0.3038	–0.3540	–0.8185	–0.4158	–0.4027	–1.1725	–0.7698
Zn – Wat_42_^*QM*^	0.497	–1.0186	–0.3040	–0.2467	–0.9357	–0.5328	–0.4028	–1.1824	–0.7796
Zn – Wat_90_^*QM*^	0.498	–0.9968	–0.3011	–0.1868	–1.0177	–0.5969	–0.4028^a^	–1.2045	–0.7837
Zn – Wat_186_^*QM*^	0.498	–0.9066	–0.3011	–0.0873	–1.0363	–0.6335	–0.4028^a^	–1.1236	–0.7208

a *E*_*xc*_^*QM*^ values taken from the IQA calculations with *n* = 42.

b The Δ*E*_*add*_^*M*^ and Δ*E*_*net*_^*M*^ values are given with respect to the gas-phase energies of the isolated M(II) cation. The total metal–water interaction energy and its classic contribution are also reported.

Table 2 illustrates the utility of the IQF descriptors to assess both the nature and relative strength of the metal–water interactions and their dependence with the size of the QM region. In principle, the metal additive energy Δ*E*_*add*_^*M*^ should converge to a fixed value as the QM region is augmented, thus reducing the impact of the QM–MM boundary effects on the energy of the central ion. Although a trend toward convergence is partly observed in our data (e.g., Δ*E*_*add*_^*Zn*(*II*)^ = −1.2383 → −1.1040 → −1.0627 → −1.0142 → −0.9968 → −0.9066 au for *n* = 0 → 6 → 18 → 42 → 90 → 186 waters in the QM region), it is clear that more QM water molecules would be required to obtain a satisfactorily converged Δ*E*_*add*_^*M*^ value. However, it is also evident that the water → metal charge transfer (Δ*q*) and the IQA terms (i.e., the net energy change Δ*E*_*net*_^*M*^ and the metal–water interaction terms) exhibit different convergence properties. Thus, the electronic density located in the metal basin achieves a nearly constant value when the QM region includes just two water shells (*n* = 18). Similarly, Δ*E*_*net*_^*M*^, which measures the stabilization of the metal ion due to charge transfer effects, and the exchange-correlation interaction between the metal ion and the surrounding waters *E*_*xc*_^*QM*^, are both well converged for the QM region with three water shells (*n* = 42). In this way, the IQA decomposition shows how the atomic description of the metal ion is essentially free from QM–MM boundary artifacts when the QM region extends up to the third solvation shell.

**Figure 2:**
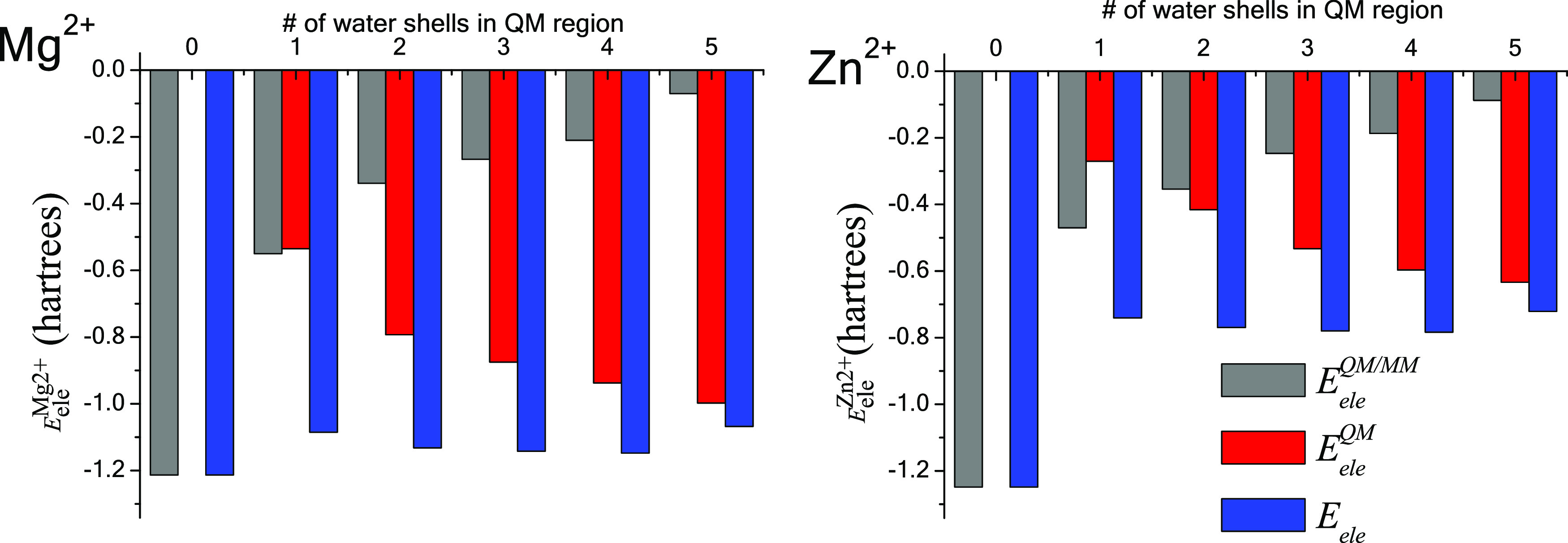
Bar diagram showing the dependence of the electrostatic interaction energy (in au) between the metal ion (*M* = Mg(II), Zn(II)) and the surrounding waters with the size of the QM region. The total, QM, and QM/MM energy components are represented by the blue, red, and gray bars, respectively.

Clearly, the lack of convergence of the Δ*E*_*add*_^*M*^ term is due to the long-range character of the electrostatic interactions between the dipositive ion and the solvent molecules as well as to the imbalance between the QM and MM descriptions. To clarify this point, the bar diagrams in Figure 2 display the evolution of the electrostatic energies with the size of the QM region. When the QM Mg(II)/Zn(II) cation sees only TIP3P waters (#shell = 0), its electrostatic interaction energy (*E*_*ele*_^*QM*/*MM*^) is maximum in absolute value. Inclusion of the first shell of water molecules in the QM region (#shell = 1) results in a fractional metal charge (*q_M_* < +2 *e*) which, in turn, reduces the magnitude of the total electrostatic term *E_ele_* = *E*_*ele*_^*QM*^ + *E*_*ele*_^*QM*/*MM*^ (see Figure 2). This loss of electrostatic stabilization is only partially compensated by gains in the net energy of the ion and by its exchange-correlation interaction with the QM waters so that the total Δ*E*_*add*_^*M*^ is actually reduced in absolute value as water molecules in the inner hydration shells enter the QM region.

Perhaps of more interest are the subtle variations in the total electrostatic energy with the size of the QM region (#shell = 1 → 2 → 3 → 4 → 5). As more waters are described quantum-mechanically, the value of *E*_*ele*_^*QM*^ increases correspondingly but is modulated by the action of two competing effects: (a) the polarization of the QM waters exerted by the central ion interactions, which is a mid-range effect with a ∼1/*r*^4^ distance-dependence (i.e., ion-induced dipole);^61^ (b) the QM water–QM water and the QM water–MM water polarization contributions, which are short-range interactions (∼1/*r*^6^; dipole-induced dipole) but of different strength because the TIP3P water molecules^62^ have a permanent dipole moment (2.37D) greater than that of the QM ones (1.92D at the B3LYP/cc-pVTZ level).

**Figure 3:**
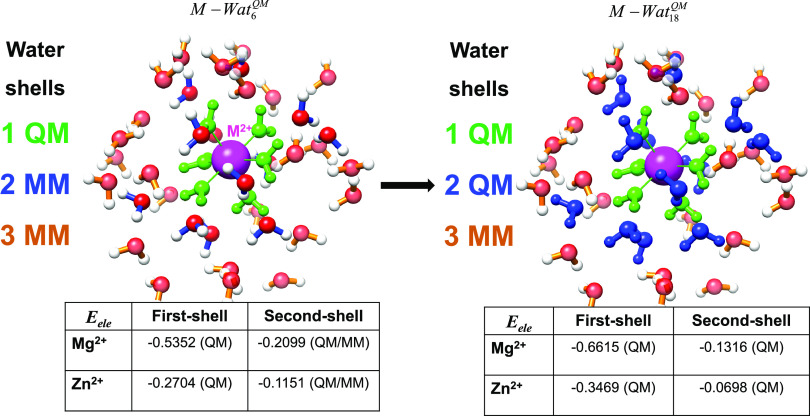
Schematic representation of the formal MM → QM conversion of the water molecules in the second shell around the M(II) cation. Electrostatic interaction energies (in au) between the metal and the first and second water shells are also indicated.

Inspection of Figure 2 shows that, for the water molecules included in the first solvation shells, mutation of MM into QM waters (e.g., #shell = 1 → 2) results in a net electrostatic stabilization. To shed light on the origin of this effect, the metal–water electrostatic energies were divided into water-shell contributions (see Figure 3 and Table S1). Thus, we see in Figure 3 that on going from the *M* – Wat_6_^*QM*^ to the *M* – Wat_18_^*QM*^ systems, the interaction between the Mg(II)/Zn(II) ion and the first-shell waters is significantly reinforced by 0.13/0.08 au, whereas the interaction with the second-shell waters, which are formally transformed from MM to QM molecules, is weakened by 0.08/0.04 au. Since the *M* – Wat_6_^*QM*^→ *M* – Wat_18_^*QM*^ conversion has a minimal effect on the metal charge, the larger electrostatic attraction between the metal ion and its first solvation shell in the *M* – Wat_18_^*QM*^ systems must arise from stronger metal–water polarization effects, that is, the first-shell waters become more polarizable by the metal ion as the QM–MM boundary is shifted outward. Reciprocally, the weaker metal–water attraction experienced by the second-shell waters located at the QM–MM boundary in *M* – Wat_18_^*QM*^ reveals the overpolarization induced by the MM waters, which is detrimental for their interaction with the central ion. Overall, the reinforced metal–water polarization in the first hydration shell dominates so that the total metal–water electrostatic energy increases when the second-shell waters enter the QM region.

The energy changes due to metal–water and water–water polarization effects along the *M* – Wat_18_ → *M* – Wat_42_^*QM*^→ *M* – Wat_90_^*QM*^ series are similar to those observed for *M* – Wat_6_ → *M* – Wat_18_^*QM*^, although the gain in the total electrostatic energy (*E*_*ele*_^*QM*^+ *E*_*ele*_^*QM*/*MM*^ in Table 2) is attenuated given that the polarization exerted by the metal ion decays with the metal–water distance. Ideally, *E*_*ele*_^*QM*^ + *E*_*ele*_^*QM*/*MM*^ would converge to a fixed value provided that the QM waters would be equally polarized by other QM or MM waters. However, this is not the case and the MM overpolarization operates at the QM–MM boundary regardless of the QM region size. This explains the significant decrease in *E*_*ele*_^*QM*^ + *E*_*ele*_^*QM*/*MM*^ for the *M* – Wat_90_ → *M* – Wat_186_^*QM*^ transition (#shell = 4 → 5; see blue bars in Figure 2) because the weak metal-induced polarization of the distant waters cannot outperform their MM overpolarization. Of course, the polarizable/non-polarizable character of the QM/MM atoms, respectively, as well as the different strengths of the QM–MM and QM–QM polarization contributions, can be considered as QM–MM artifacts. Hence, our analysis shows that the IQA/IQF additive energies are suitable indicators to investigate in detail the energetic impact of QM–MM boundary artifacts and the convergence of local energetic properties with respect to the size of the QM system.

The IQA terms in Table 2 and Figure 2 help outline the similarities and differences in the nature of the Mg(II)/Zn(II)–water interactions. We see that the water → metal charge transfer (Δ*q*) is mainly due to the inclusion of the first hydration shell in the QM region and is more pronounced (as expected) for the softer Zn(II) ion. For example, the values of Δ*q* = 0.209 *e^–^* (Mg) and 0.493 *e^–^*(Zn) in the *M* – Wat_6_^*QM*^ systems are only slightly below those for the *M* – Wat_42_^*QM*^ systems, 0.212 and 0.497 *e^–^*, respectively. The patterns in Δ*q* are translated into the magnitude and stabilization of the net energies of the metal ions (Δ*E*_*net*_^*M*^), as well as in those of the short-range *E*_*xc*_^*QM*^ energies. As mentioned above, both Δ*E*_*net*_^*M*^ and *E*_*xc*_^*QM*^ achieve nearly converged values at #shell = 3, and the Mg/Zn ratios of their limiting values reveal that the impact of charge transfer effects is about 3 times larger for the Zn(II) cation than for Mg(II). Moreover, for the Zn(II) cation, the magnitude of the purely electrostatic interaction energy is approximately twice that of the exchange-correlation contribution, but the equivalent ratio for Mg(II) is significantly higher at ∼10. Such dominant role of electrostatics as measured by the IQA energy decomposition gives support to the use of nonbonded MM potentials (i.e., Coulombic plus Lennard–Jones terms) for representing the Mg(II)–water interactions*.* In contrast, the IQA analysis suggests that more sophisticated nonbonded potentials capturing charge transfer effects may be required for representing the Zn(II)-water interactions. As a matter of fact, we note that the IQA descriptors might be employed to develop more accurate potentials as those that have been inspired by other EDAs.^5^

### QM/MM Calculations on the MMP-12/Inhibitor Complexes

**Figure 4:**
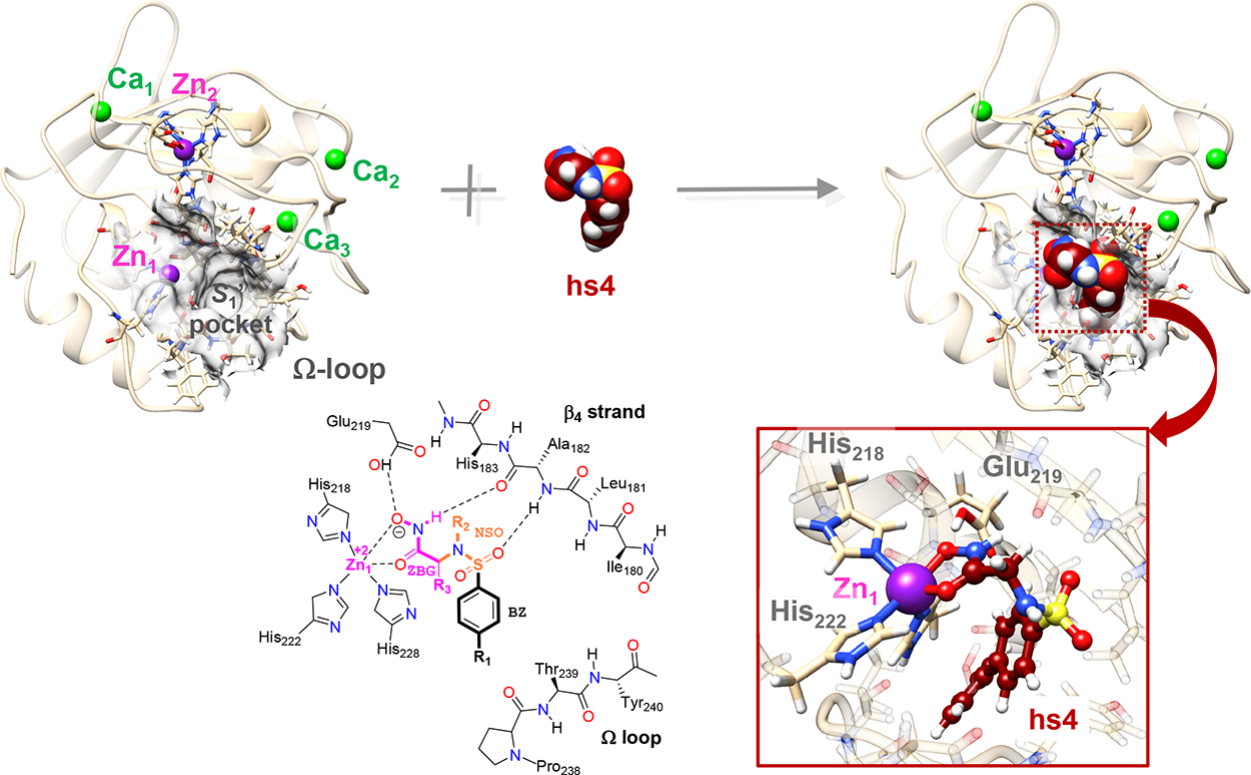
Ribbon representation of the 3F17 crystallographic structure (after molecular edition) with and without the inhibitor molecule. Specific residues within the catalytic region are in the stick model. The Ca (green) and Zn (purple) ions as well as the inhibitor molecule are in CPK representation. The transparent surface characterizes the S_1_′ pocket. In the inset (bottom right), the coordination environment of the catalytic Zn ion is shown in detail with the inhibitor atoms in ball-and-stick representation. Schematic representation (bottom left) of a generic inhibitor bound within the active site showing the ZBG (C atoms in magenta), the NSO (Cs in orange), and the BZ (Cs in black) defined for the IQF analyses.

As mentioned above, the homologous MMP-12 inhibitors studied in this work share a common ZBG (hydroxamate) and a benzene-sulfonamide moiety as shown in Figure 4. In the crystallographic structures, the catalytic zinc ion (Zn_1_) chelates the ZBG in a bidentate manner. In addition, the ZBG establishes H-bond contacts with the carboxylic group of the catalytically important Glu_219_ residue and the backbone CO group of Ala_182_, which is included in the β4-strand of residues that constitute other binding spots for peptide substrates. The sulfonamide group of the inhibitors also interacts with the β4-strand, while its hydrophobic moiety (-Ph-R_1_) is placed within the so-called S_1_′ pocket, which largely determines the metalloenzyme specificity for substrates and ligands (see Figure 4). The various ligand substituents provide additional H-bond sites and/or modulate the hydrophobic binding to the S_1_′ pocket. For the purpose of carrying out the EDA of the QM/MM energies, each inhibitor molecule is split into three different fragments corresponding to the ZBG group (including the -R_3_ substituent), the sulfonamide group (-N(*R*_2_)-SO_2_- denoted as NSO), and the benzene ring with its para-substituent (−R_1_) denoted as BZ in Figure 4.

The structural differences among the various MMP-12/inhibitor complexes are small as shown by the computed root-mean-square deviations (RMSD), ranging between 0.3 and 0.6 Å (see Table 3). These RMSD values were calculated for the heavy atoms in selected active site residues and in the ligand skeleton with respect to those of the MMP-12/hs7 complex (see Figure 4). Hence, the placement of the inhibitor molecule in the active site is very similar in all the MMP-12/inhibitor complexes.

**Table 3:** Energy Changes (Δ*E*^*QM*/*MM*^ Coulombic and vdW, Δ*G*_*solv*_^*PBSA*^ Solvation; in kcal/mol) and QM/MM–PBSA Scorings (Δ*G*) for the Interaction between the Enzyme and Ligand on the Partially Relaxed X-ray Structures^a^

	hs7	hs1	hs3	hs4	hs5	hs6	nhk	z79
RMSD (Å)	0.00	0.29	0.50	0.56	0.31	0.35	0.58	0.53
Δ*E*^*QM*/*MM*^(*Coul*)	–311.7 (9.9)	–327.1 (9.6)	–307.3 (9.5)	–319.5 (9.5)	–313.7 (9.8)	–315.0 (10.2)	–323.2 (9.5)	–318.4 (10.3)
Δ*E*_*IQA*_^*QM*/*MM*^(*Coul*)	–307.4 [4.3]	–326.8 [0.3]	–305.0 [2.3]	–324.0 [4.5]	–312.8 [0.9]	–313.4 [1.6]	–321.1 [2.1]	–316.9 [1.5]
Δ*E*^*QM*/*MM*^(*vdW*)	–37.5	–51.3	–48.6	–49.4	–40.4	–40.5	–49.4	–45.4
Δ*G*_*solv*_^*PBSA*^	255.9	274.3	255.9	263.1	258.0	260.4	269.1	263.5
Δ*G*	–93.3	–104.1	–100.0	–105.8	–96.0	–95.1	–103.4	–100.3

a Δ*E*_*IQA*_^*QM*/*MM*^ stands for the IQA-reconstructed value. Values in parentheses correspond to the counterpoise correction of the basis set superposition error in the QM/MM energies. Values in squared brackets correspond to the estimation of the IQA numerical error (i.e., |*ΔE*^*QM*/*MM*^(*Coul*) – Δ*E*_*IQA*_^*QM*/*MM*^(*Coul*)|). Root-mean-square deviations (in Å) of the MMP-12/inhibitor structures with respect to the MMP-12/hs7 complex are also given.

Table 3 collects the binding energy contributions derived from the QM/MM calculations using the D3-B3LYP/cc-pVTZ(−*g*) level of theory for the QM region and the AMBER force field for the MM atoms. The sum of the QM/MM binding energy and the PBSA solvation energy yields the QM/MM–PBSA ranking of the MMP-12/inhibitor complexes (Δ*G* values in Table 3), which are reasonably correlated with the binding free energies Δ*G_exp_* obtained from isothermal calorimetry measurements (the determination coefficient *R^2^* has a value of 0.85, the Spearman correlation coefficient being 0.84; see the correlation plot in Figure S1). When comparing the gas-phase Δ*E*^*QM*/*MM*^ with the experimental data, we find a worse correlation (*R^2^* = 0.45), thus showing the importance of complementing the QM/MM energies with solvation free energies. For the sake of comparison, we also tested the performance of a similar QM/MM–PBSA approach using the semiempirical DFTB3 Hamiltonian^63,64^ as well as that of purely MM–PBSA calculations, resulting in worse *R^2^* values of 0.45 and 0.02, respectively (see the Supporting Information for further details). Hence, although computationally expensive, the D3-B3LYP/cc-pVTZ(−*g*) QM/MM–PBSA energies seem to capture the basic trends in the binding of benzene-sulfonamide inhibitors with the MMP-12 enzyme.

Concerning the relative weight of intermolecular contacts and solvation effects, the enzyme–ligand attraction in vacuum (−378 < Δ*E*^*QM*/*MM*^(*Coul*) + Δ*E*^*QM*/*MM*^(*vdW*)< −349 kcal/mol) overcompensates the accompanying desolvation penalty (256 < Δ*G_solv_* < 274 kcal/mol). The large magnitude of these energy terms shows the considerable abundance and strength of the enzyme–inhibitor contacts. We estimated the basis set superposition error (BSSE) inherent to the QM relative energies using the counterpoise method,^65^ but the resulting BSSE values were minimal (<3%). Hence, the resulting Δ*G* values (Δ*G* = Δ*E*^*QM*/*MM*^ + Δ*G_solv_* ∼ – 90/–100 kcal/mol) are much lower than the experimental binding energies, which lie in a narrow range between −9.9 and −11.8 kcal/mol. Several factors contribute to explain this fact: the lack of entropy contributions, the neglect of enzyme/inhibitor relaxation, the unbalanced description of enthalpic and solvation effects, and the exaggeration of the electrostatic interaction energy due to QM–MM overpolarization. This latter factor seems particularly important because the closely related MM–PBSA approach leads to Δ*G* values between −23 and −42 kcal/mol that, although uncorrelated with the experimental data, are much smaller in absolute value. Nonetheless, in terms of the affinity ranking, the predictive capacity of the B3LYP-based QM/MM–PBSA scoring (*R^2^* = 0.85) justifies the interest of performing the EDA study using the IQA method.

To assess the numerical errors in the IQA calculations that arise in the construction of atomic basins and in the numerical integration operations, Table 3 includes both the *ΔE*^*QM*/*MM*^(*Coul*) and Δ*E*_*IQA*_^*QM*/*MM*^(*Coul*) energies. The latter values are reconstructed from the various IQA terms that are directly obtained from numerical integration over the atomic basins, thus excluding the dispersion and PBSA empirical contributions. Unfortunately, the underlying IQA numerical errors are not systematic so that Δ*E*_*IQA*_^*QM*/*MM*^(*Coul*) can be above or below Δ*E*^*QM*/*MM*^(*Coul*). Nevertheless, taking into account the large magnitude of the QM/MM relative energies (>300 kcal/mol in absolute value), the mean unsigned difference between Δ*E*^*QM*/*MM*^(*Coul*) and Δ*E*_*IQA*_^*QM*/*MM*^(*Coul*) remains within reasonable bounds (2.2 kcal/mol). Hence, this figure can be taken as an upper bound to the uncertainty in the various IQA/IQF descriptors (the dispersion and PBSA energies are not affected by the IQA numerical errors since they are not obtained through numerical integration of the QM charge density). We also note in passing that the minimal BSSE effects are not considered in the IQA analysis owing to important problems in the real-space partitioning associated to the ghost basis set functions.^66^

The decomposition of the QM/MM–PBSA energies gives rise to a myriad of atomic and diatomic energy contributions, which are conveniently grouped into fragment terms corresponding to amino acid residues, metal ions and the three inhibitor fragments defined in Figure 4. For the sake of simplicity, it is appropriate to focus on the IQF decomposition of the Δ*G* scorings in terms of additive energies, which, in turn, are derived from the partitioning of the individual energies (*G* = *E^QM^* + *E*_int_^*QM*/*MM*^ + *G_solv_*) for the complex and separate enzyme/inhibitors according to eqs 12 and 16. The resulting IQF descriptors (Δ*G*_add_ in Table 4), which differ substantially in several kcal/mol even though the MMP-12/inhibitor structures superimpose closely with RMSD below 0.6 Å, assess the relative weight of the various residues in the enzyme–inhibitor affinity. Thus, it turns out that the largest favorable contribution is always due to the inhibitor sulfonamide group (NSO in Table 4; ranging from −28 to −53 kcal/mol) followed by the catalytic Zn_1_ ion and its His ligands, which exhibit varying contributions within −3 and −19 kcal/mol depending on the inhibitor structures. The hydrophobic tail of the inhibitors (i.e., the BZ group) has a significant contribution (ca. −5 and −21 kcal/mol), while the nearby β4 and Ω-loop enzyme residues (e.g., Ala_182_, Tyr_240_, etc.) are stabilized by a few kcal/mol.

**Table 4:**
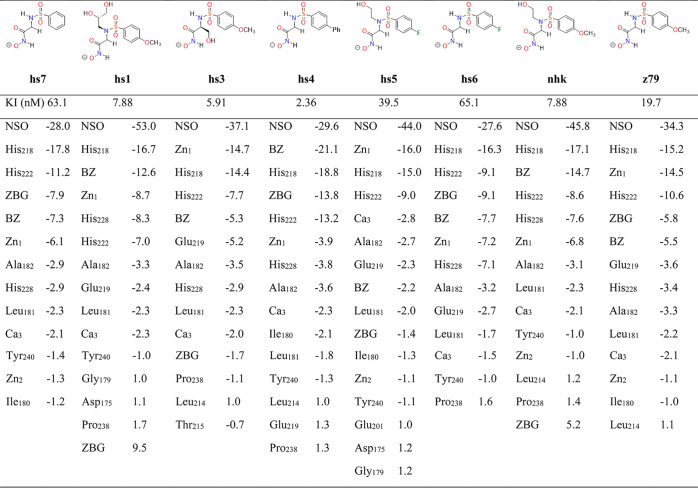
IQF-Based Additive Contributions (Δ*G_add_* in kcal/mol) to the QM/MM PBSA Scoring of the Most Important Residues/Fragments

A closer inspection of data in Table 4 reveals that the IQF additive energy of the ZBG, which binds directly to the Zn(II) metal, is either moderately stabilizing (e.g., −8 kcal/mol in the MMP-12/hs7 complex) or repulsive (e.g., +9 in MMP-12/hs1). Actually, the largest pairwise IQF interaction term is associated to the Zn_1_···ZBG (classical and exchange-correlation) interaction, the ZBG inhibitor experiencing the largest electronic charge reduction (Δ*q* ≈ 0.16 *e*^–^) upon complexation. For example, the *E*_int_^Zn1···ZBG^ values are −246.6, −258.9, −252.3, −246.0, −251.6, −248.1, −258.5, and −251.6 kcal/mol for the MMP12 complexes with hs7, hs1, hs3–hs6, nhk, and z79, respectively (these and other *E*_int_^*IJ*^ values are available at the output files uploaded to the data repository; see below). Nonetheless, this and other strong interactions of the ZBG group are largely compensated by its equally important desolvation penalty and electronic deformation, which are measured by the ZBG contribution to the PBSA solvation (i.e., Δ*G*_*solv*_^ZBG^ ≈ 80 kcal/mol) and its fragment net energy (i.e., Δ*E*_*net*_^ZBG^ ∼90 kcal/mol), respectively (see Table 5). In particular, the high value of Δ*E*_*net*_^ZBG^ is indicative of the ZBG → enzyme charge transfer, although the overall charge rearrangement affects the His ligands (Δ*q* ≈ −0.10 *e*^–^) more than the Zn_1_ ion (Δ*q* ∼ 0.05 *e*^–^). Of course, the ZBG plays an essential role in determining the inhibitor binding mode, but the other inhibitor fragments may have a larger energetic impact according to the IQF analysis.

**Table 5:** IQF Components (in kcal/mol) Associated to the Three Fragments Constituting the Inhibitor Structures^a^

	hs7	hs1	hs3	hs4	hs5	hs6	nhk	z79
ZBG								
Δ*q*^ZBG^	0.170	0.165	0.176	0.140	0.145	0.178	0.169	0.173
Δ*E*_*net*_^ZBG^	91.6	93.1	97.8	87.5	86.6	87.5	94.6	91.2
Δ*G*_*solv*_^ZBG^	72.5	91.7	77.2	69.3	85.6	74.6	86.4	78.8
Δ*E*_int_^ZBG^/2	–172.1	–175.3	–176.8	–170.6	–173.5	–171.2	–175.9	–175.8
NSO								
Δ*q*^NSO^	0.052	0.081	0.054	0.052	0.073	0.053	0.074	0.047
Δ*E*_*net*_^NSO^	–6.3	–6.0	–2.2	–8.8	–6.6	–4.1	–6.0	–5.9
Δ*G*_*solv*_^NSO^	22.5	4.7	14.1	21.9	9.7	20.7	6.6	20.1
Δ*E*_int_^*NSO*^/2	–44.3	–51.8	–49.0	–42.6	–47.1	–44.2	–46.4	–48.5
BZ								
Δ*q*^BZ^	0.086	0.077	0.070	0.129	0.130	0.068	0.075	0.095
Δ*E*_*net*_^BZ^	23.5	24.6	30.8	14.5	15.2	29.5	26.7	31.8
Δ*G*_*solv*_^BZ^	–9.6	–10.4	–5.0	–12.5	–12.5	–8.8	–10.8	–8.4
Δ*E*_int_^BZ^/2	–21.2	–26.8	–31.1	–23.5	–24.3	–28.5	–30.5	–28.8

a The changes in the electron population (Δ*q*) are also indicated.

Concerning the sulfonamide group, we find a characteristic IQF fingerprint dominated by favorable binding contributions (see Table 5). The presence of a bifurcated -NSO_2_···NH H-bond with Ala_182_/Leu_181_ induces a negative change in the NSO net energy upon complexation (Δ*E*_*net*_^NSO^, −2 and −9 kcal/mol). It is also a considerably polar and polarizable group, which results in large electrostatic attractions with the nearby metal ions located at 6–7 Å away (the NSO···Zn_1_ and NSO···Ca_3_ pairwise energies have values around −30 and −48 kcal/mol, respectively). These through-space interactions determine an overall IQF interaction energy of Δ*E*_int_^NSO^ around −40/–50 kcal/mol depending on the inhibitor species, which, in turn, dominate the largely negative additive IQF energies of the NSO group in Table 4. Although we recognize that these IQF descriptors for NSO are likely exaggerated due to the underlying QM–MM overpolarization effects, they suggest a twofold role for this group by promoting both the H-bond interactions with the β4 strand and through-space electrostatic attractions with metal ions.

To gain further insight into the substituent effects, several comparisons between pairs of MMP-12 complexes can be made. On one hand, the energetic impact of the R_2_ substituents, which is absorbed into the IQF quantities of NSO, occurs mainly by reducing the solvation energy change of the expanded NSO fragment rather than by reinforcing (or giving new) enzyme–inhibitor contacts (e.g., Δ*G*_*solv*_^NSO^ = 20.7 → 9.7, Δ*E*_int_^NSO^ = −44.2 → −47.1, and Δ*E*_*net*_^NSO^ = – 4.1 → −6.6 kcal/mol for *R*_2_ = H → CH_2_CH_2_OH comparing the hs6 and hs5 complexes; similar trends arise in nhk/z79 and hs1/hs3; see Table 5). On the other hand, the *para*-substitution at BZ with a nonpolar hydrophobic group (hs4 with R_1_ = Phe; *K*_I_ = 2.36 nM) increases notably the enzyme inhibition as compared with that of the parent structure (hs7 with R_1_ = H, *K*_I_ = 61.6 nM), resulting also in a deeper burial of the inhibitor molecule within the S_1_′ pocket. The MMP-12/hs7 → MMP-12/hs4 comparison in terms of the IQF descriptors points out that the BZ additive contribution (Δ*G*_*add*_^BZ^ = –7.3 → −21.5 kcal/mol) explains most of the larger affinity, the rest of the IQF descriptors being less influenced. The gains in Δ*G*_*add*_^BZ^ arise from stronger vdW interactions (−9.0 → −15.7) as well as from an attenuated fragment electronic deformation (23.5 → 14.2). The equivalent *para*-substitution with electron-withdrawing groups (-F, -OCH_3_) reinforces both the vdW and Coulombic interactions of the −BZ moiety with the close residues (see Table 5). However, they are essentially canceled by larger fragment deformation energies (e.g., Δ*E*_int_^BZ^ = −21.2 → −28.5 and Δ*E*_*net*_^BZ^ = 23.5 → 29.5 kcal/mol for *R*_1_ = H → F comparing the hs6 and hs7 complexes). As a matter of fact, the fluorinated hs6 inhibitor (*K*_I_ = 65.1 nM) ranks very closely to hs7 (*K*_I_ = 61.6), what seems in consonance with the minimal changes in their IQF descriptors (see Table 4). However, incorporation of the *para*-methoxy substituent intensifies inhibitor binding (*K*_I_ = 19.7 for z79), and according to the IQF analysis, this would be a secondary effect ascribed to the sulfonamide moiety (NSO), whose Δ*G*_*add*_^NSO^ changes from −28.0 (MMP-12/hs7) to −34.3 (MMP-12/z79) kcal/mol, mainly as a consequence of more favorable interactions and desolvation. These changes are in line with the slightly bigger *e*^–^ population (+0.005 *e*^–^) of the NSO fragment in the MMP-12/z79 complex, thus showing the sensitivity of the fragment IQF energies to subtle electronic effects.

## Summary and Conclusions

The extension of the IQA methodology presented in this work is a step forward toward the detailed energy decomposition of large biomolecular systems described with QM/MM methods, thus yielding valuable information about energy changes at the residue level. Taking advantage of the IQA characteristics as a real-space energy decomposition that splits the QM energy into atomic and diatomic contributions, we have shown that the QM–MM electrostatic interaction is readily included as one more pairwise IQA term. Moreover, the QM–MM vdW energy together with the effective atomic solvation energies extracted from PBSA calculations are easily incorporated into the IQA framework. It is thus feasible to partition QM/MM–PBSA scorings for all kinds of receptor–ligand complexes or other systems.

For the two cases of study considered in this work under the prism of the QM/MM or QM/MM–PBSA IQA calculations, we have obtained interesting results that highlight the utility of the EDA methodologies as applied to macromolecular systems. On one hand, thanks to the careful analysis of fragment-based interaction and net energies in the selected metal–water clusters, it has been possible to detect and monitor the underlying unbalance between QM–QM and QM–MM interactions, which is commonly assumed to result in the overpolarization of the QM region. In this way, it turns out that IQA descriptors may help in the diagnosis of QM/MM methodological problems and in the evaluation of possible solutions. On the other hand, we have shown that the gas-phase QM/MM calculations on the structurally similar MMP-12/inhibitor complexes must be complemented with PBSA solvation estimates in order to produce binding scorings that correlate reasonably well with the experimental data. The QM/MM–PBSA scorings largely overestimate the experimental binding energies (due in part to QM–MM overpolarization issues, as well as the lack of entropy contributions and the neglect of distortion and proton rearrangement effects upon inhibitor binding). Anyway, focusing on the relative trends, their IQA-based partitioning unveils a complex interplay of intra- and interfragment/solvation effects due to the small electronic rearrangements occurring within the QM region upon complexation. Nevertheless, the IQA descriptors are able to score the binding relevance of each enzyme and/or inhibitor residue in the examined complexes and to explain the source of their binding contributions.

Finally, we note that the routine application of the QM/MM IQA calculations is still challenging owing to the high computational cost of the numerical construction of the interatomic surfaces delimiting the atomic basins and the six-dimensional integrations within each basin and pair of basins. Furthermore, as suggested by our analysis of the MMP-12/inhibitors, the QM/MM IQA component energies are prone to experience significant variations upon small geometric/electronic changes, and therefore, conformational sampling may be required. Given that some numerical uncertainty is inherent to the IQA approach, the nature of the molecular system under study should be considered so that, for instance, the dissection of weak noncovalent binding effects may be problematic. However, regardless of these difficulties, which will be overcome thanks to continuous improvements in computational algorithms and hardware technologies, our results clearly support the QM/MM IQA strategy as the most suitable EDA in order to achieve a smooth and fruitful energy decomposition for QM systems embedded within MM atomistic potentials and solvent continuum.

## Supporting Information Available

(Table S1) IQF energy components for the interaction between metal ions and hydration shells; (Table S2) test IQA calculations with the cc-pVTZ(−*g*) and cc-pVTZ basis sets; (Figure S1) correlation plots between QM/MM–PBSA and MM–PBSA scorings and experimental data (PDF)

## Notes

All the IQA QM/MM data analyzed in this work can be downloaded at the Mendeley data repository (doi: 10.17632/f69z2gbgdp.1).

## Notes

The modular version of the PROMOLDEN code together with a set of auxiliary BASH and Octave scripts that are required for performing QM/MM IQA calculations are available upon request.
