# Supplementary material for: QM/MM Energy Decomposition Using the Interacting Quantum Atoms Approach
Source: J Chem Inf Model. 2022 Feb 25;62(6):1510–24. doi: 10.1021/acs.jcim.1c01372 (PMC8965874; doi:10.1021/acs.jcim.1c01372)
Supplement: Supplementary file 1 — ci1c01372_si_001.pdf [file ci1c01372_si_001.pdf]

SUPPORTING INFORMATION

QM/MM Energy Decomposition using the Interacting Quantum Atoms Approach

Roberto López[(a)], *Natalia Díaz*[(b)], *Evelio Francisco*[(b)],

Ángel Martín-Pendás[(b)] *and Dimas Suárez*[(b)]*

(a) Departamento de Química y Física Aplicadas. Universidad de León. Facultad de Biología. Campus de Vegazana s/n. 24071 León (Castilla y León) Spain.

(b) Departamento de Química Física y Analítica. Universidad de Oviedo. Facultad de Química. Julián Clavería 8. 33006 Oviedo (Asturias) Spain.

Table S1. IQF energy components (in Hartrees) for the interaction between the metal ion and the hydration shells for each QM regions considered (X^{2+}, $X-Wat_{6}^{QM}$, $X-Wat_{18}^{QM}$, $X-Wat_{42}^{QM}$, $X-Wat_{90}^{QM}$, $X-Wat_{186}^{QM}$ where X = Mg(II), Zn(II)) in the metal-water clusters studied in this work.

M···(waters 1 to 6) First-shell

**QM Subsystem**	$E_{ele}^{QM/MM}$	E_{int}^{QM}	E_{ele}^{QM}	E_{xc}^{QM}
Mg^{2+}	-0.5998	-	-	-
$Mg-Wat_{6}^{QM}$	-	-0.6746	-0.5352	-0.1394
$Mg-Wat_{18}^{QM}$	-	-0.8018	-0.6615	-0.1403
$Mg-Wat_{42}^{QM}$	-	-0.8152	-0.6747	-0.1405
$Mg-Wat_{90}^{QM}$	-	-0.6760	-0.6760	-0.1405
$Mg-Wat_{186}^{QM}$	-	-0.6763	-0.6763	-0.1405
Zn^{2+}	-0.6249	-	-	-
$Zn-Wat_{6}^{QM}$	-	-0.6716	-0.2704	-0.4012
$Zn-Wat_{18}^{QM}$	-	-0.7486	-0.3469	-0.4017
$Zn-Wat_{42}^{QM}$	-	-0.7630	-0.3614	-0.4016
$Zn-Wat_{90}^{QM}$	-	-0.3629	-0.3629	-0.4016
$Zn-Wat_{186}^{QM}$	-	-0.3630	-0.3630	-0.4016

M···(waters 7 to 18) Second-shell

**QM Subsystem**	$E_{ele}^{QM/MM}$	E_{int}^{QM}	E_{ele}^{QM}	E_{xc}^{QM}
Mg^{2+}	-0.2344	-	-	-
$Mg-Wat_{6}^{QM}$	-0.2099	-	-	-
$Mg-Wat_{18}^{QM}$	-	-0.1319	-0.1316	-0.0003
$Mg-Wat_{42}^{QM}$	-	-0.1682	-0.1679	-0.0003
$Mg-Wat_{90}^{QM}$	-	-0.1942	-0.1942	-0.0003
$Mg-Wat_{186}^{QM}$	-	-0.1949	-0.1949	-0.0003
Zn^{2+}	-0.1526	-	-	-
$Zn-Wat_{6}^{QM}$	-0.1151	-	-	-
$Zn-Wat_{18}^{QM}$	-	-0.0699	-0.0689	-0.0010
$Zn-Wat_{42}^{QM}$	-	-0.1050	-0.1040	-0.0010
$Zn-Wat_{90}^{QM}$	-	-0.1192	-0.1192	-0.0010
$Zn-Wat_{186}^{QM}$	-	-0.1187	-0.1187	-0.0010

Table S1 (cont.)

M···(waters 19 to 42) Third-shell

QM Subsystem	$E_{ele}^{QM/MM}$	E_{int}^{QM}	E_{ele}^{QM}	E_{xc}^{QM}
Mg^{2+}	-0.0804	-	-	-
$Mg-Wat_{6}^{QM}$	-0.0720	-	-	-
$Mg-Wat_{18}^{QM}$	-0.0719	-	-	-
$Mg-Wat_{42}^{QM}$	-	-0.0326	-0.0325	-0.0001
$Mg-Wat_{90}^{QM}$	-	-0.0451	-0.0451	-0.0001
$Mg-Wat_{186}^{QM}$	-	-0.0548	-0.0548	-0.0001
Zn^{2+}	-0.1427	-	-	-
$Zn-Wat_{6}^{QM}$	-0.1074	-	-	-
$Zn-Wat_{18}^{QM}$	-0.1071	-	-	-
$Zn-Wat_{42}^{QM}$	-	-0.0678	-0.0675	0.0003
$Zn-Wat_{90}^{QM}$	-	-0.0853	-0.0853	0.0003
$Zn-Wat_{186}^{QM}$	-	-0.1016	-0.1016	0.0003

M···(waters 43 to 90) Fourth-shell

QM Subsystem	$E_{ele}^{QM/MM}$	E_{int}^{QM}	E_{ele}^{QM}	E_{xc}^{QM}
Mg^{2+}	-0.0636	-	-	-
$Mg-Wat_{6}^{QM}$	-0.0569	-	-	-
$Mg-Wat_{18}^{QM}$	-0.0568	-	-	-
$Mg-Wat_{42}^{QM}$	-0.0568	-	-	-
$Mg-Wat_{90}^{QM}$	-	-0.0219	-0.0219	0.0000
$Mg-Wat_{186}^{QM}$	-	-0.0516	-0.0516	0.0000
Zn^{2+}	-0.0796	-	-	-
$Zn-Wat_{6}^{QM}$	-0.0600	-	-	-
$Zn-Wat_{18}^{QM}$	-0.0599	-	-	-
$Zn-Wat_{42}^{QM}$	-0.0598	-	-	-
$Zn-Wat_{90}^{QM}$	-	-0.0295	-0.0295	0.0000
$Zn-Wat_{186}^{QM}$	-	-0.0587	-0.0587	0.0000

Table S1 (cont.)

M···(waters 91 to 186) Fifth-shell

QM Subsystem	$E_{ele}^{QM/MM}$	E_{int}^{QM}	E_{ele}^{QM}	E_{xc}^{QM}
Mg^{2+}	-0.0741	-	-	-
$Mg-Wat_{6}^{QM}$	-0.0664	-	-	-
$Mg-Wat_{18}^{QM}$	-0.0663	-	-	-
$Mg-Wat_{42}^{QM}$	-0.0663	-	-	-
$Mg-Wat_{90}^{QM}$	-0.0663	-	-	-
$Mg-Wat_{186}^{QM}$	-	-0.0201	-0.0201	0.0000
Zn^{2+}	-0.0498	-	-	-
$Zn-Wat_{6}^{QM}$	-0.0375	-	-	-
$Zn-Wat_{18}^{QM}$	-0.0374	-	-	-
$Zn-Wat_{42}^{QM}$	-0.0374	-	-	-
$Zn-Wat_{90}^{QM}$	-0.0374	-	-	-
$Zn-Wat_{186}^{QM}$	-	-0.0083	-0.0083	0.0000

Table S2. Test IQA calculations in the $Zn-Wat_n^{QM}$ systems with the cc-pVTZ(-*g*) and cc-pVTZ basis sets. The examined descriptors are the Zn ion the water→metal charge transfer (Δq in e^-), change of the IQF additive atomic energies (ΔE_{add}^{M}, in au) and energy components (ΔE_{net}^{M}, $E_{ele}^{QM/MM}$, E_{int}^{QM}, E_{ele}^{QM}, E_{xc}^{QM}, in au) for the Zn(II) ion and its interaction with the surrounding waters in the $Zn-Wat_n^{QM}$ systems with *n*=6 and 18. The ΔE_{add}^{M} and ΔE_{net}^{M}, values are given with respect to the gas-phase energies of the isolated Zn(II) cation.

QM Subsystem	Δq	ΔE_{add}^{M}	ΔE_{net}^{M}	$E_{ele}^{QM/MM}$	E_{int}^{QM}	E_{ele}^{QM}	E_{xc}^{QM}	E_{int}^{QM} + $E_{ele}^{QM/MM}$	E_{ele}^{QM} + $E_{ele}^{QM/MM}$
				**cc-pVTZ(-g) for Zn**					
$Zn-Wat_6^{QM}$	0.493	-1.1083	-0.3025	-0.4700	-0.6716	-0.2704	-0.4012	-1.1416	-0.7404
$Zn-Wat_{18}^{QM}$	0.497	-1.0670	-0.3038	-0.3540	-0.8185	-0.4158	-0.4027	-1.1725	-0.7698
				**cc-pVTZ for Zn**					
$Zn-Wat_6^{QM}$	0.493	-1.1097	-0.3040	-0.4700	-0.6716	-0.2704	-0.4012	-1.1416	-0.7404
$Zn-Wat_{18}^{QM}$	0.497	-1.0686	-0.3052	-0.3540	-0.8188	-0.4161	-0.4027	-1.1727	-0.7701

The data in Table S2 indicate that the impact of the *g* functions on the IQA descriptors is minimal. The largest effect arises in the change of net atomic energies (~0.0015 hartrees) while the interaction terms are hardly affected. We note also that the computational cost of the IQA calculations is nearly doubled upon inclusion of the *g* functions.

Figure S1 Comparison between the calculated Δ*G* energies (in kcal/mol) and the experimental binding free energies. The determination coefficient (R^2), the Spearman correlation coefficient (ρ) and the root mean square error of the relative differences (RMS, in kcal/mol) are indicated.

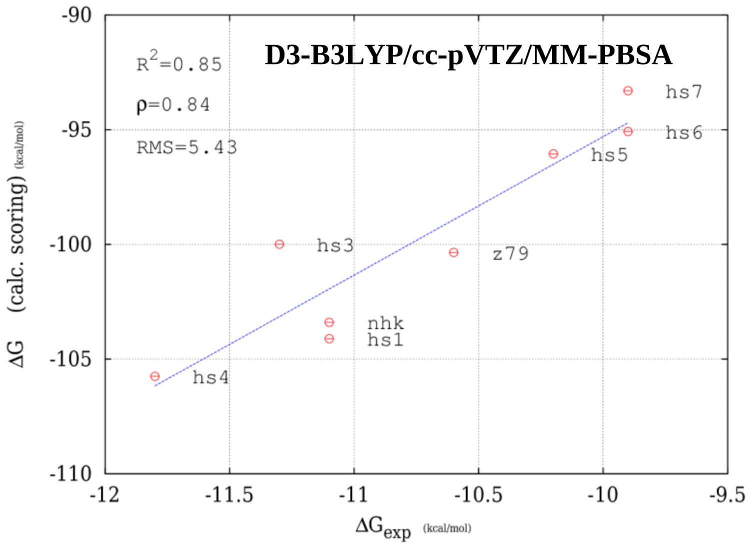

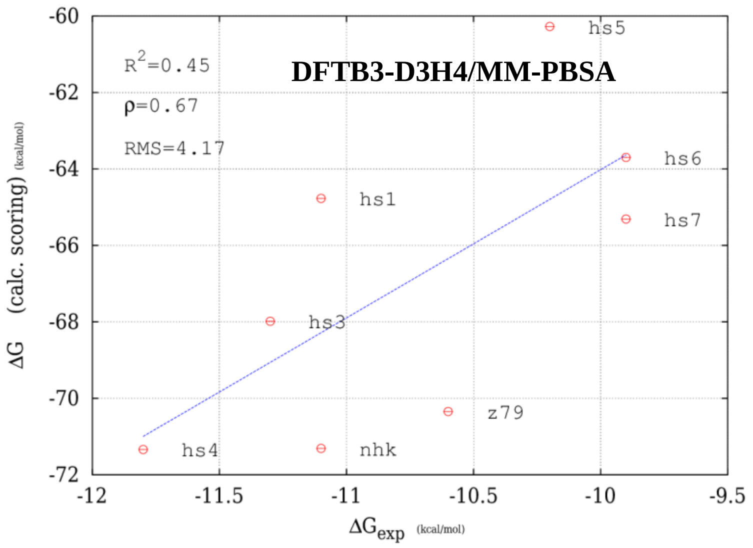

Figure S1 (cont.)

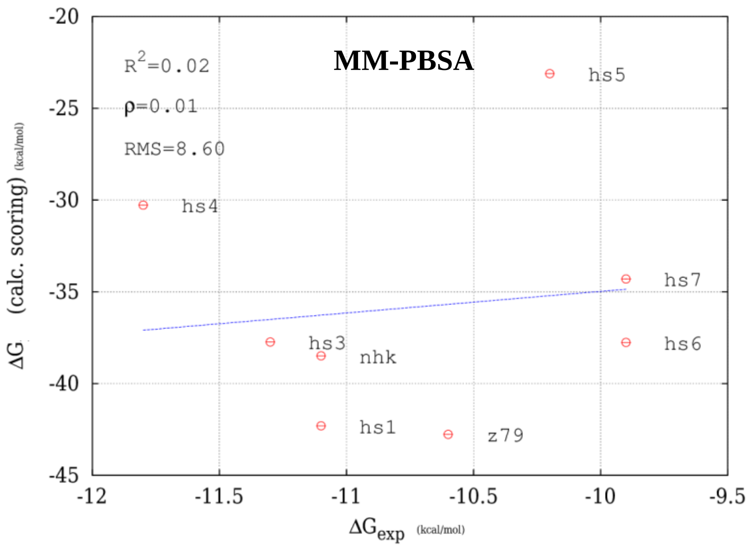

The QM/MM calculations with the DFTB3 Hamiltonian were carried out using analogous settings to those employed with the D3-B3LYP/cc-pVTZ(-g) level of theory (*e.g.*, identical MM representation and QM region selection). More particularly, we used the DFTB3 version of the self-consistent charges density functional tight-binding (SCC-DFTB) method,[1, 2] in combination with the Slater-Koster parameters extracted from the 3OB set.[3, 4] The geometry of the QM region was relaxed by means of QM/MM geometry optimization with the *sander* program. Subsequently, the gas-phase DFTB3/MM energies were complemented with dispersion and hydrogen-bonding corrections[5] evaluated with the Cuby4 framework,[6] as well as with the PBSA solvation energy term, which was computed using the DFTB3 Mulliken charges for the QM atoms. The same DFTB3/MM geometries were employed to carry out single-point MM-PBSA calculations on the MMP12/inhibitor structures.

1. Elstner, M.; Porezag, D.; Jungnickel, G.; Elsner, J.; Haugk, M.; Frauenheim, T.; Suhai, S.; Seifert, G., Self-Consistent-Charge Density-Functional Tight-Binding Method for Simulations of Complex Materials Properties. *Phys. Rev. B* **1998**, *58*, 7260-7268.
2. Krüger, T.; Elstner, M.; Schiffels, P.; Frauenheim, T., Validation of the Density-Functional Based Tight-Binding Approximation Method for the Calculation of Reaction Energies and other Data. *J. Chem. Phys.* **2005**, *122*, 114110.
3. Gaus, M.; Goez, A.; Elstner, M., Parametrization and Benchmark of DFTB3 for Organic Molecules. *J. Chem. Theory Comput.* **2013**, *9*, 338-354.
4. Kubillus, M.; Kubař, T.; Gaus, M.; Řezáč, J.; Elstner, M., Parameterization of the DFTB3 Method for Br, Ca, Cl, F, I, K, and Na in Organic and Biological Systems. *J. Chem. Theory Comput.* **2015**, *11*, 332-342.
5. Pecina, A.; Haldar, S.; Fanfrlík, J.; Meier, R.; Řezáč, J.; Lepšík, M.; Hobza, P., SQM/COSMO Scoring Function at the DFTB3-D3H4 Level: Unique Identification of Native Protein–Ligand Poses. *J. Chem. Inf. Model.* **2017**, *57*, 127-132.
6. Grimme, S.; Hansen, A.; Brandenburg, J. G.; Bannwarth, C., Dispersion-Corrected Mean-Field Electronic Structure Methods. *Chem. Rev.* **2016**, *116*, 5105-5154.
